# Advances in the Prevention of Infection-Related Preterm Birth

**DOI:** 10.3389/fimmu.2015.00566

**Published:** 2015-11-16

**Authors:** Ronald F. Lamont

**Affiliations:** ^1^Research Unit of Gynecology and Obstetrics, Department of Gynecology and Obstetrics, Institute of Clinical Research, Odense University Hospital, University of Southern Denmark, Odense, Denmark; ^2^Division of Surgery, University College London, London, UK

**Keywords:** infection, antibiotics, bacterial vaginosis, preterm labour/labor, preterm birth

## Abstract

Infection-related preterm birth (PTB) is more common at early gestational ages and is associated with major neonatal mortality and morbidity. Abnormal genital tract microflora in early pregnancy predicts late miscarriage and early PTB. Accordingly, it is logical to consider antibiotics as an intervention. Unfortunately, the conclusions of systematic reviews and meta-analyses (SR&MAs) carried out in an attempt to explain the confusion over the heterogeneity of individual studies are flawed by the fact that undue reliance was placed on studies which: (a) had a suboptimal choice of antibiotic (mainly metronidazole) or used antibiotics not recommended for the treatment of bacterial vaginosis (BV) or BV-related organisms; (b) used antibiotics too late in pregnancy to influence outcome (23–27 weeks); and (c) included women whose risk of PTB was not due to abnormal genital tract colonization and hence unlikely to respond to antibiotics. These risks included: (a) previous PTB of indeterminate etiology; (b) low weight/body mass index; or (c) detection of fetal fibronectin, ureaplasmas, Group B streptococcus or *Trichomonas vaginalis*). While individual studies have found benefit of antibiotic intervention for the prevention of PTB, in meta-analyses these effects have been negated by large methodologically flawed studies with negative results. As a result, many clinicians think that any antibiotic given at any time in pregnancy to any woman at risk of PTB will cause more harm than good. Recently, a more focused SR&MA has demonstrated that antibiotics active against BV-related organisms, used in women whose risk of PTB is due to abnormal microflora, and used early in pregnancy before irreversible inflammatory damage has occurred, can reduce the rate of PTB. This review presents those data, the background and attempts to explain the confusion using new information from culture-independent molecular-based techniques. It also gives guidance on the structure of putative future antibiotic intervention studies.

## The Importance of Preterm Birth

In high-income countries, preterm birth (PTB), particularly at early gestations, is the major cause of death and handicap in neonates (1–3). Babies born at 22, 24, and 26 completed weeks of gestation have an infant mortality rate of 54, 21, and 2%, respectively, and a rate of survival without major morbidity at 365 days of 0.02, 14.1, and 45.9%, respectively (2). Approximately 65% of babies born between 22 and 26 completed weeks of gestation will die on the labor ward or in the neonatal intensive care unit and at 30-month follow-up, around 50% will be handicapped and in 50% of these, the handicap will be severe. Accordingly, at 2.5 years of age only 12–13% will be alive and intact (3). In the UK, the cost of hospital readmissions in the first five and 10 years of life is 20 times greater for those babies born before 28 completed weeks of gestation compared to those born after 37 completed weeks (4). In 2007, the Institute of Medicine calculated that the annual cost associated with PTB in the USA was $26.2 billion comprising medical costs for the baby ($16.9 billion), labor and delivery costs for the mother ($1.9 billion), early intervention programs for children with disabilities and developmental delays from birth to age 3 years ($611 million), special education services ($1.1 billion), and lost work and pay for those born preterm ($5.7 billion) (5). It has been demonstrated that between 23 and 26 completed weeks of gestation, each day of prolongation of pregnancy increases the survival rate by 3% (6).

## Infection as a Cause of Preterm Birth

Spontaneous preterm labor (SPTL) leading to PTB is now recognized as a syndrome caused by a number of pathological processes leading to activation of the common terminal pathway of parturition (7). The etiology of SPTL is multifactorial but there now exists abundant evidence that local or systemic infection or inflammation is a major cause, particularly of early PTB (8–10). This involves *inter alia*, prostaglandins (PGs), proinflammatory chemokines and cytokines, as well as pattern recognition receptors known as toll-like receptors (11, 12). In addition, the relationship between infection and PTB changes as pregnancy progresses. Infection in late PTB (34–36 weeks) is unusual but is present in most cases in which PTB occurs before 30 weeks gestation (13). In addition, the earlier in pregnancy at which PTB occurs, the more likely it is to be due to infection (14, 15). Between 26 and 34 completed weeks of gestation, women admitted in SPTL are more likely to have abnormal genital tract microflora and chorioamnionitis compared to women delivered electively at the same gestational age for feto-maternal indications (16–18). Compared with term birth, the prevalence of maternal endometritis, chorioamnionitis and neonatal infection is much more common following PTB (10, 13, 19, 20). The gestational age association of acute chorioamnionitis shows a dramatic reduction from 94.4% at 21–24 weeks to 39.6% (25–28 weeks), 35.4% (29–32 weeks), 10.7% (33–36 weeks), and only 3.8% at 37–40 weeks (21).

## The Prediction of Infection-Related Preterm Birth

As pregnancy progresses, the genital tract microflora becomes progressively more benign such that by term; the vaginal microflora poses no significant threat to the fetus as it passes through the birth canal (22). Although all births before 37 completed weeks of gestation are defined as preterm, PTB before 32 weeks gestation (2% of all births) accounts for most of the neonatal mortality and morbidity (23). Accordingly, if screening and treatment begins at gestations beyond 24 weeks, the opportunity to prevent late miscarriage and very early PTB is lost. The main cohort studies from Europe, North America, and Indonesia (24–33) and three case control studies from the USA, Sweden, and Australia (34–36) have used different methodologies to examine the association between abnormal genital tract microflora either in the form of bacterial vaginosis (BV) or the presence of BV-associated organisms and adverse outcomes of pregnancy. The majority of these studies show a statistically significant association between abnormal genital tract microflora and late miscarriage and PTB. Furthermore, the degree of risk is greater the earlier in pregnancy at which abnormal microflora was detected (24). A positive screening test for abnormal genital tract microflora at 26–32 weeks gestation is associated with a statistically significant 1.4- to 1.9-fold increased risk of PTB (27, 29–32). In contrast, a positive result from screening in the second trimester is associated with a 2.0- to 6.9-fold increased risk of an adverse outcome (25–28). In a longitudinal study of women in Indonesia, women with BV in early pregnancy had a 21% risk of an adverse outcome, compared to only 11% of those who developed the condition later in pregnancy (27).

### Abnormal Genital Tract Microflora

Due to the polymicrobial nature of vaginal microflora, the definition of what is normal or abnormal genital tract microflora is very difficult. Normal vaginal microflora is assumed to be present in the absence of disease. Disease results from the interplay between microbial virulence, numerical dominance, and the innate and adaptive immune response of the host. Disease is assumed to be absent if the woman is asymptomatic, and there are no clinical signs of vaginal infectious morbidity. Abnormal vaginal microflora may occur (a) because of a sexually transmitted infection; (b) colonization by an organism which is not normally a constituent part of the vaginal microbial community such as *Haemophilus influenzae*, or *Listeria monocytogenes*; (c) due to increased virulence or overgrowth of an organism that is normally a constituent part of the vaginal microflora, e.g., *Escherichia coli*; or (d) BV.

### The Bacterial Vaginosis Syndrome

Disordered vaginal microflora sometimes now referred to as “dysbiosis” is most commonly due to BV – a polymicrobial condition, characterized by a significant decrease in the quantity or quality of lactobacilli in association with a 1,000-fold increase in the number of other potentially pathogenic organisms such as *Gardnerella vaginalis*, *Mycoplasma hominis*, *Mobiluncus* species, and other anaerobic organisms. Since many of the organisms associated with BV are quite fastidious, and since BV is a quantitative rather than a qualitative change in vaginal microflora, qualitative or semiquantitative culture techniques are unhelpful for diagnosis. Accordingly, the diagnosis of BV requires quantification of vaginal microbiota (37). With the introduction of culture-independent techniques, more sensitive and specific ways of diagnosing BV may be developed (38–40). BV affects almost a third of women (41). In gynecological practice, BV has been found to be associated with the acquisition of STIs such as chlamydia, gonorrhea, trichomoniasis, and viral infections (HIV, HSV, and HPV), plus a range of morbidities including postabortal sepsis, infertility, pelvic inflammatory disease, and posthysterectomy vaginal cuff infections. In pregnancy, BV has been associated with PTB, preterm prelabor rupture of the membranes (PPROM), early, late, and recurrent miscarriage, and postpartum endometritis (24). If BV is detected early in pregnancy, it is associated with a five to sevenfold increased risk of SPTL and PTB (25, 26). A longitudinal study in pregnancy has demonstrated that only 2% of women who did not have BV in the second trimester will develop BV by 34 weeks. In contrast, 50% of women who had BV in the second trimester will still have BV at 34 weeks (42).

#### New Information from Culture-Independent, Molecular-Based Techniques

Recent evidence from cultivation-independent molecular-based techniques has demonstrated that BV is not a single entity but a syndrome (the BV syndrome) of different sub-types with different etiologies, different microbial communities, and hence different responses to antibiotics and, in all likelihood, different subsequent phenotypical outcomes from normal term birth to late miscarriage, very early PTB, PPROM, or preterm stillbirth (38). Such new information clarifies why the etiology remains unknown, why the microbiology of BV differs from case to case, and why the response to antibiotics remains inconsistent. It would also explain why the phenotypic outcome of pregnancy differs from case to case, ranging from a normal outcome to a very early PTB or late miscarriage. A better understanding would help to limit the administration of antibiotics for the prevention of infection-related PTB to those antibiotics that are known to be effective in women with objective evidence of abnormal vaginal microflora and use these antibiotics early in pregnancy before inflammation and tissue damage has occurred (43).

## Antibiotics for the Prevention of Infection-Related Preterm Birth

This review reflects the use of prophylactic antibiotics used early in pregnancy for the prevention of PTB and does not cover the management of PPROM, which may be a cause, or a result of infection and probably a combination of the two. The literature pertaining to the use of antibiotics following PPROM is legion and so may be the subject of a separate review.

We know that abnormal vaginal colonization in early pregnancy is predictive of PTB (24, 25, 27–36). Accordingly, it is logical to consider the use of antibiotics for the prevention of infection-related PTB. Unfortunately, antibiotic studies have chosen different: (a) risk groups; (b) diagnostic methods; (c) degrees of abnormal vaginal colonization; (d) antibiotic dose regimens and routes of administration; (e) women with different host susceptibilities and hence host response; (f) gestational age at time of treatment; (g) outcome parameters; and (h) definitions of success (44–62). Understandably, the results of such studies are conflicting. A number of systematic reviews and meta-analyses (SR&MAs) of these studies have been conducted and updated (63–74). However, SR&MAs are retrospective analyses of pooled data that are only as good as the quality of studies included (75). Due to the aforementioned limitations of the studies published to date, the conclusions derived from these SR&MAs are also limited and should not be used to provide guidelines or make recommendations for the use or change of practice (75). Similarly, until recently, none of the SR&MAs on the use of antibiotics for the prevention of infection-related PTB has simultaneously addressed the optimal choice of agent, the choice of patient, and the timing of intervention. If antibiotic intervention is to be successful in reducing the incidence of PTB, these antibiotics (a) should be active against those organisms known to be associated with PTB, (b) should only be used in women with abnormal genital tract microflora, and (c) should be used early in pregnancy before infection and inflammation have had an opportunity to cause irreversible damage which will inevitably lead to SPTL and PTB.

### Choice of Antibiotic

The Centers for Disease Control and Prevention (CDC) do not recommend erythromycin or coamoxiclav for the treatment of BV. Their recommendation is to use either metronidazole or clindamycin, orally or vaginally (76). Like macrolide antibiotics, clindamycin has anti-inflammatory properties (77–81) and has a broader range of activity against BV-related organisms such as species of *Mobiluncus* and the genital mycoplasmas (82–87). Metronidazole and other nitro-imidazoles are inactive *in vitro* against BV-associated organisms such as *M. hominis*, *G. vaginalis*, *Ureaplasma urealyticum* (88, 89), and *Atopobium vaginae* (90, 91). In addition, they have little or no activity against other aerobic organisms such as *Staphylococcus aureus* or species of *Streptococci*. However, metronidazole has a similar treatment success rate as clindamycin *in vivo* (76). This suggests one or both of two possible mechanisms. Firstly, *in vivo*, BV-related organisms may be sensitive to the hydroxy-metabolite of metronidazole. Alternatively and more likely, metronidazole acts indirectly by destroying anaerobes which provide nutrients to other BV related organisms such as *G. vaginalis* or *A. vaginae* (92). Molecular-based studies have indicated a far greater diversity of microorganisms associated with BV than has been evident from culture-dependent techniques (38). These organisms form different communities that may be anaerobe dominated or *G. vaginalis* and *A. vaginae* dominated. Other abnormal subtypes may be due to mixed organisms or perhaps due to a subtype caused by sexual transmission (38). Accordingly, it is possible that those sub-types of BV in which anaerobes are dominant are more successfully treated by metronidazole. In contrast, in other subtypes where anaerobic organisms are not dominant, metronidazole may be less effective. Finally, clindamycin may be active against both metronidazole-sensitive sub-types but also against a wider range of BV sub-types with different microbial communities. It should be noted that while *M. hominis* is extremely sensitive to clindamycin, *U. urealyticum* is only weakly sensitive to clindamycin (87, 93).

#### Effect on Lactobacilli

When comparing clindamycin with metronidazole, the case for metronidazole and against clindamycin is often given that metronidazole conserves vaginal lactobacilli, whereas clindamycin destroys them. However, phage virus colonization of lactobacilli is associated with BV, and it has been postulated that diet acquired phage viruses, may be induced to become lytic by a factor related to sexual activity, or alternatively that *Lactobacillus* phages may be directly inoculated into the vagina from sexual partners (94). If phage virus colonization of lactobacilli is present, metronidazole may be perpetuating rather than curing BV, whereas the opposite would be true with clindamycin.

#### PREMEVA1 Trial

In 2013, an abstract presented orally to the Society for Maternal–Fetal Medicine was published on-line. http://dx.doi.org/10.1016/j.ajog.2013.10.036. The PREMEVA1 trial was a French multicentre randomized controlled trial comprising 2,869 low-risk women randomized to receive clindamycin or placebo before 15 weeks’ gestation. In the placebo group, late abortion/very preterm spontaneous delivery rate (12–32 weeks) did not differ significantly between the clindamycin and placebo groups. Requests for details of the study have elicited no response. Accordingly, at the time of completion of this manuscript, no peer-reviewed, full-study report could be found on any of the appropriate search engines. This being the case, the risk of bias cannot be assessed and, until the details of the study are fully available, it is difficult to comment on the significance of the findings and these should not be used to influence guidelines.

#### Route of Administration of Clindamycin

The choice between oral clindamycin or clindamycin vaginal cream (CVC) to treat abnormal genital tract microflora/BV in pregnancy needs to be addressed. Vaginal administration is the most direct and efficient route of administration of antibiotic to the site of the heaviest bacterial load. In contrast, we know that BV is associated with subclinical endometritis (95). Accordingly, if vaginal microorganisms have already gained access to the choriodecidua, they may not be treatable by CVC, and systematic therapy may be necessary. To the authors knowledge, no study has studied the simultaneous combined use of CVC and oral clindamycin.

#### The Potential for Newer Macrolide Antibiotics

More data are now available on azithromycin and a new antibiotic, solithromycin, that may be considered candidate antibiotics in future intervention studies. In a SR&MA, macrolides and clindamycin administered during the second trimester of pregnancy were associated with a reduction in the rate of PTB (96). Second trimester metronidazole used alone was associated with an increased risk of PTB in a high-risk population. Like many other SR&MAs, studies were included where the risk of PTB was positive fetal fibronectin, urogenital mycoplasma infection, previous PTB of unqualified phenotype, or prepregnancy weight of <50 kg. In addition, while azithromycin and clarithromycin were included in the search, the only macrolide included was erythromycin (96). In a RCT of interconceptional antibiotics to prevent PTB, neither azithromycin nor metronidazole was of any benefit in reducing the subsequent rate of PTB (97).

Two recent studies from Malawi tested the effect of prophylactic azithromycin on the subsequent rate of PTB. In a high-risk population, routine prophylaxis with azithromycin showed no benefit, but this population was unselected (high risk of poor pregnancy outcome but not specifically PTB), and no objective evidence of infection-related risk of PTB was sought (98). Also in Malawi, a RCT of intermittent treatment of maternal malaria and reproductive tract infection with monthly sulfadoxine-pyrimethamine plus two doses of azithromycin was associated with a significant reduction in PTB and low birth weight (99).

Solithromycin is a new antibiotic that is highly potent against ureaplasmas and mycoplasmas and other antibiotic resistant organisms. In an animal study, combined intra-amniotic (IA) and intravenous administration of solithromycin resulted in effective concentrations of solithromycin in amniotic fluid (AF) and maternal and fetal plasma, leading the authors to conclude that solithromycin may have promise in future for the prevention of PTB (100). Subsequent studies showed that a 4-day course of solithromycin eradicated IA *Ureaplasma parvum* infection in the same sheep model (101).

### Choice of Patient

Women with BV (Nugent score 7–10) respond better to clindamycin than women with intermediate microflora (Nugent score 4–6). Accordingly, prophylactic antibiotics to prevent infection-related PTB should only be given to women with objective evidence of abnormal vaginal colonization such as BV (37). Without such evidence, treatment may disrupt, rather than treat, abnormal microflora. In many antibiotic intervention studies, the indication for administration of prophylactic antibiotics was previous PTB. There is no doubt that previous PTB is a known risk factor for subsequent PTB (102, 103). However, a previous PTB may have been for feto-maternal indications, such as antepartum hemorrhage and fulminating pre-eclampsia. Such indications would not place a subsequent pregnancy at risk of infection-related PTB and consequently are unlikely to benefit from antibiotic prophylaxis. Such studies (104), have been erroneously cited as evidence that antibiotics have no role in the prevention of PTB (105). Only a small portion of such women (even those with BV) may be at risk for PTB. Better diagnostic or predictive methods are required to improve our ability to identify those women who would be most likely to benefit from treatment with antibiotics.

#### Pharmacogenetics

Pharmacogenetics is also an important consideration (106). Metronidazole used in mainly Black or Hispanic women in North America has not shown benefit (52). In contrast, in predominantly White North European women, five studies using clindamycin have shown benefit (43). Black or Hispanic women may have a genetic predisposition to mount a damaging inflammatory response to the challenge of BV, while predominantly white Northern European women do not. Alternatively, in predominantly white Northern European women, the inflammatory response may be sufficiently less rigorous to allow time for antibiotic therapy to be of benefit. Finally, it may be that some women with a prior PTB have a genetically non-infectious risk of PTB under which circumstances their propensity to deliver preterm will exist with or without BV and hence would not be expected to respond to antibiotic treatment. These studies and the findings of racial differences in the vaginal microbiome highlight the importance of tailoring antibiotic treatment approaches for different racial groups and controlling for race in clinical trials.

#### Treatment of Symptomatic or Asymptomatic Pregnant Women with BV

Symptomatic pregnant women with BV should be treated even if they are at otherwise low risk of PTB. The management of asymptomatic women with BV who do not have other risk factors such as a previous BV-related PTB is less well accepted. Since BV is an independent risk factor for PTB, one could argue that any woman with BV (symptomatic or asymptomatic) is at a significant twofold increased risk of PTB, if BV is detected at or beyond 24 completed weeks of gestation. This is the same risk associated with smoking which is considered significant enough to merit intervention. In contrast, if BV is detected before 16 weeks gestation, there is a five to sevenfold increased risk of PTB (see the Section on The Prediction of Infection-Related Preterm Birth). If one relies on previous PTB as a risk factor for subsequent PTB in women with BV, it is essential to record the phenotype of that previous PTB. If the previous PTB was iatrogenic, because of twins, APH, or pregnancy-induced hypertension/preeclampsia, it may not be relevant in women with BV. Similarly, if the previous PTB was unexplained apart from a maternal weight <50 kg or a BMI <18 kg/m^2^ then the detection of BV may be irrelevant. As more information becomes available from molecular-based, cultivation-independent techniques, the identification of sub-types of BV, the different etiologies of each, the different microbiology, the different response to antibiotics, and the different phenotypic outcomes may address this concern.

### Timing of Antibiotics

Abnormal genital tract microflora in early pregnancy, even if this reverts to normal, is still associated with late miscarriage and PTB (44) suggesting that whatever damage is done by infection and inflammation, this occurs early and persists (44, 107–113). If antibiotics are used late in pregnancy when inflammatory tissue damage may have already occurred, and there are already irreversible changes in the cervix, myometrium, decidua, placenta, and extraplacental membranes, then antibiotics are unlikely to be of benefit. Accordingly, concern has been expressed that under these circumstances, antibiotics may cause more harm than good (114–120). Hence, it may be argued that antibiotics should be used early in pregnancy before infection/inflammation can cause irreversible damage that ultimately leads to SPTL and PTB. In their recommendations for the treatment of BV in pregnancy, the CDC treatment guidelines advise the use of oral or vaginal metronidazole or oral clindamycin (76). However, it was noted that the late administration of CVC up to 32 weeks gestation was associated with subsequent adverse outcomes, such as low birth weight and neonatal infection (28, 48, 51). As a result, the guidelines recommend that CVC should only be used in the first half of pregnancy (76).

#### Gene–Environmental Interaction

Many diseases like PTB are due to a combination of genetic susceptibility and environmental exposure. A woman may have the environmental exposure (BV), but if she does not have the genetic susceptibility (gene polymorphism) to mount a damaging inflammatory response then little harm may occur. Conversely, a woman may possess the gene polymorphism to mount a damaging inflammatory response, but if she does not have environmental exposure (BV) then damage may not occur. However, when both susceptibility and exposure are present, the risk of an adverse outcome will be increased, and this is referred to as the gene–environmental interaction (121). Abnormal vaginal microflora or infection leads to adherence, invasion, and host inflammatory response. That response may be appropriate resulting in tissue repair and healing. Alternatively, the response may be exaggerated (hyper-response) resulting in tissue damage from increased production and release of proinflammatory cytokines. Conversely, the response may be inadequate (hyporesponse) leading to overwhelming infection. Both a hyper-response and a hypo-response may result in mortality and morbidity due to tissue damage. If antibiotics are used late in this process, it may not be possible to prevent irreversible tissue damage, morbidity, and mortality. In contrast, if antibiotics are used early, before tissue damage occurs, this damage might be prevented. Accordingly, the earlier the gestational age at which clindamycin is administered to women with objective evidence of risk of infection-related PTB, the more likely it is to be able to demonstrate a reduction in the rate of PTB (43).

#### Potential for the Use of Anti-inflammatory Agents as Adjunctive Treatment

The potential for adding to antibiotics an anti-inflammatory agent which targets the NF-κB and p38 MAPK (cytokine suppressive anti-inflammatory drugs [CSAIDs]) that block cytokine signaling for the prevention and treatment of inflammation-induced PTB shows promise and has been comprehensively reviewed elsewhere (122). In an ovine model, IA administration of a single dose of CSAID suppressed the lipopolysaccharide-induced IA inflammatory response with minimal fetal effects (123). Several animal model studies have shown additional benefit of antibiotic cotreatment with anti-inflammatory agents. In the rhesus monkey, following IA inoculation of *U. parvum*, azithromycin plus dexamethasone and indomethacin was able to prolong pregnancy and prevent advanced fetal lung injury (124). Similarly, to determine whether treatment with ampicillin/dexamethasone/indomethacin (AMP/DEX/INDO) delayed PTB induced by IA Group B streptococcus (GBS) inoculation in rhesus monkeys, ampicillin alone eradicated GBS but uterine activity, AF cytokines, PGs, and matrix metalloprotein (MMP)-9 remained elevated. In contrast, the combination of AMP/DEX/INDO suppressed interleukin-1β, TNF-α, PGE_2_, and PGF_2α_ but did not alter MMP expression or chorioamnionitis. The combination of AMP/DEX/INDO suppressed inflammation and significantly prolonged gestation (125).

### Rescreening and Retreating with Antibiotics

In many antibiotic intervention studies there has been inconsistency of rescreening and retreatment in which persistent or recurrent BV occurs in ~10–30% (43). Using stringent diagnostic criteria (BV on Nugent score together with all four elements of Amsel’s clinical composite criteria), 70.8% of women who received CVC were cured/improved at 20–24 days post-treatment compared to only 12% in the placebo group. Recurrence rates in those CVC patients successfully treated were ~6% at 6 weeks postbaseline and 10% at 28–34 weeks. Of the 29.2% of women who failed to respond to the first 3-day course of CVC and who were therefore retreated with a 7-day course of CVC, 32.6% and 51.2% were cured/improved at 20–24 days postretreatment and at 28–34 weeks gestation, respectively (126). Accordingly, rescreening and retreating in pregnancy may be helpful since an initial course of CVC cured or improved BV in 88% of women, and a second course some 3–6 weeks later was still able to cure or improve BV in 50% of those who still had the condition (127).

## Critical Review of the Literature on the Use of Antibiotics to Prevent Infection-Related Preterm Birth

As discussed earlier, the majority of the SR&MA that consider the use of antibiotics for the prevention of PTB inappropriately merged clindamycin and metronidazole studies together rather than considering them separately. Those studies that initially considered clindamycin and metronidazole studies separately then erred by combining the two antibiotics when considering the gestational age at treatment (63, 66, 74). While the majority of studies included in the SR&MA comprised women with objective evidence of BV, two meta-analyses included studies where the risk-status or entry criteria was measured by other parameters unrelated to BV. These included parameters, such as positive fetal fibronectin test, previous PTB, and detection of GBS, *U. urealyticum*, or trichomonas (66, 74). Using these SR&MAs, it can be concluded that if inappropriate antibiotics are used at late gestations, in women without objective evidence of abnormal vaginal bacterial colonization, there is no benefit with respect to the prevention of infection-related PTB. However, concerns have been expressed that if these SR&MAs are not interpreted carefully, they will be erroneously cited as evidence that any antibiotic, given to any pregnant woman, at any gestational age will be unhelpful in preventing PTB. For this interpretation, caution has been urged (114–120, 128–130). Two large studies are regularly cited in SR&MA as evidence that antibiotics are of no benefit for the prevention of PTB: the National Institutes for Child Health and Human Development (NICHD)/Maternal Fetal Medicine Network Units (MFMU) study (52) and the ORACLE II study (131, 132). Despite their faults (see below), these studies markedly outweigh all other studies in SR&MA and hence strongly influence conclusions.

### The NICHD/MFMU (2000) Study

This study screened 29,626 women (52) of which 6,540 were positive solely for BV without other conditions, such as trichomoniasis. From these, the recruitment was low with only 1,936 (29.6%) randomized to receive either metronidazole or placebo. Of the 4,604 exclusions, 999 were excluded for reasons recorded as “other.” Most SR&MAs classify this study as having been in a “low-risk population” yet 85% of the population was either Black or Hispanic. As a part of the methodology, up to 8 weeks could elapse between screening and initiation of treatment. During this delay, the grade of microflora on Gram stain changed in 25% of women (115). Metronidazole was administered as a once only 2 g oral dose and unsurprisingly, vomiting occurred in a high percentage of women. Under such circumstances, the 2 g oral dose was repeated 2 days later. With no objective measure of compliance, such as metronidazole blood levels, the number of women who took the repeat course remains undocumented. Importantly, there was an inexplicable 37% placebo effect while BV remained in 22% of the metronidazole group and 63% of the placebo group at 1 month. This suggests confounding by factors, such as a lack of effectiveness of metronidazole in the treatment group or spontaneous resolution in the placebo group. Finally, treatment was started late in pregnancy with 44% treated after 20 weeks gestation, and no women were treated before 16 weeks gestation.

### ORACLE II Study

The ORACLE II trial is commonly cited as evidence that antibiotic treatment does not prevent PTB. More accurately, the ORACLE II trial should be cited as demonstrating that if inappropriate antibiotics are given to women too late in pregnancy with no objective evidence of abnormal vaginal microflora then they are ineffective in preventing infection-related PTB. Accordingly, in any SR&MAs related to the use of antibiotics to prevent infection-related PTB, the Oracle II study should be excluded; sadly, this is not the case and due to the numbers involved the study has a strong weighting such that the positive results of other studies are negated. Erythromycin and coamoxiclav were used in this study (132) because of the perceived importance of *Ureaplasmas* in neonatal infectious morbidity (133), but neither is recommended for the treatment of BV (76). It is notable that erythromycin, while being effective against *Ureaplasma* spp., exhibits minimal passage across the placenta and hence does not reach effective concentrations in AF. Hence it is not effective in eradicating intrauterine *Ureaplasma* infections. Women with known infections were excluded, and the trial protocol required no objective evidence of abnormal vaginal colonization for the diagnosis of BV (37). Without objective evidence of abnormal vaginal microflora, at least 60% and probably more at this late gestation were not in infection-related SPTL. There are also serious concerns about the accuracy of diagnosis of SPTL. Only 50% of cases required tocolytics and around 90% of women were still undelivered after 48 h. Approximately 85% of women remained undelivered by 7 days and the mean gestational age at delivery was 38 weeks. The timing of administration of antibiotics was also questioned since the intervention occurred after SPTL had begun. Finally, in the 7-year follow-up report of the ORACLE II study (131), the assessment of cerebral palsy has been questioned. The assessment only applied to the two-thirds of cases that were recruited from the UK rather than the Republic of Ireland, and the assessment of cerebral palsy was based upon telephone calls to the parents, and in some cases, parent completed postal questionnaires rather than an objective, structured neurobehavioral assessment by skilled healthcare professionals.

### Cochrane Systematic Review (Updated 2013)

The recently updated Cochrane Review (63, 74) is already being cited as evidence that antibiotics are unhelpful for the prevention of PTB. The updated review (74) is extensive and contains data from 21 trials and reports 57 different analyses. These numbers are necessary because the included studies used different risk groups, diagnostic methods, degrees of abnormal microflora, antibiotic dose regimens and routes of administration, host susceptibilities, host response, gestational age at time of treatment, outcome parameters, and definitions of success (44–53, 55–58) resulting in different results. However, in contrast to the systematic review reported below (43), the Cochrane Review (74) includes studies which used antibiotics that are not recommended for the treatment of BV and importantly did not consider the effect of pharmacogenetics (106) as outlined in the Section above “Choice of patient.” In addition, the review included women with a previous PTB of non-infectious etiology. It did not differentiate between clindamycin and metronidazole when assessing the benefit of treatment before 20 weeks gestation. The review selected studies of pregnant women with either “BV” or “intermediate microflora” without considering that these are different entities with differing rates of response to antibiotics (134). Finally, the review did not include recent evidence of the benefit of rescreening and retreating BV in pregnancy (127) and the title of the Review was “the treatment of bacterial vaginosis in pregnancy” and should not have been used to comment on the prevention of preterm birth.

### AJOG Systematic Review 2011

To address the deficiencies of existing SR&MA with respect to the optimal choice of agent, the choice of patient, and the timing of intervention, we performed a SR&MA of clindamycin use before 22 weeks gestation in women with abnormal genital tract microflora (43). The hypothesis of the review was that previous SR&MA on the use of antibiotics used prophylactically for the prevention of PTB or their individual studies were flawed by the fact that undue reliance was placed on studies in which suboptimal antibiotics (mainly metronidazole) were used. They were also flawed by the fact that antibiotics were used too late in pregnancy to influence outcome (23–27 weeks gestation) and used in women whose risk of PTB was not due to BV but due to some other markers not directly related to infection. Conversely, the hypothesis of the SM&MA was that antibiotics that are active against BV or BV-related organisms that are appropriately used in women whose risk of PTB is due to abnormal genital tract colonization and that are administered early in pregnancy before irreversible inflammatory damage occurs can reduce the rate of PTB. The primary outcome of the studies included in this SR&MA was spontaneous PTB at <37 completed weeks gestation and late miscarriage. These were chosen because they were used in most meta-analyses that evaluated preventative strategies for PTB. In the meta-analysis, the RR for delivery <33 weeks was 0.44 (95% CI: 0.41–1.41; nine versus four cases), but due to the low numbers this was not statistically significant. Although the reduction was consistent with the beneficial effect of clindamycin seen in the later gestational age groups, further research is required to confirm efficacy at lower gestations. The SR&MA demonstrated that when clindamycin was compared to controls, administration before 22 weeks gestation to women with objective evidence of abnormal genital tract microflora was associated with a significant reduction in the rates of PTB and late miscarriage by 40 and 80%, respectively.

#### Secondary Outcome Variables

The secondary outcome variables demonstrated that of those infants born preterm, low birth weight occurred in 20% of those who received clindamycin compared to 80% of those who received no treatment (*P* < 0.009). There was also a 32.5-day difference in the mean prolongation of pregnancy in favor of clindamycin compared with no treatment (*P* < 0.024) (62). In women with the highest Nugent Score of 10, late miscarriage and PTB occurred in 5.4% of those who received clindamycin compared to 35.7% of those who received placebo (60). Finally, the rate of late miscarriage or PTB was 28% in those women with persistent BV compared to 10% in those in whom BV was cured (OR = 2.9; 95% CI = 1.3–5.2), and the rate of late miscarriage or PTB was 15% in women with cured but recurrent BV, compared to only 2% in those women whose BV was cured with no recurrence (OR = 9.3; 95% CI = 1.6–53.5) (54).

## Safety of and Resistance to Antibiotics in Pregnancy

The safety of antimicrobials in pregnancy has recently been reviewed (135). A common response to the case for antibiotic use to prevent infection-related PTB is that we already use antibiotics too frequently in pregnancy. However, few can cite local/personal audit of such practice. Accordingly, we reported a large, population-based study comprising nearly 1 million Danish women which demonstrated that >40% received antimicrobials at some stage during pregnancy (136). We felt that this might be an underestimate because Denmark, like other Nordic countries, is cautious about the use of antibiotics in pregnancy. In addition, the Registry used included only antibiotics obtained by prescription in the community. Our response would be that by employing a more focused approach to the use of antibiotics for the prevention of infection-related PTB we would, in effect, be reducing the indiscriminate use of antibiotics already demonstrated. The development and introduction of new antibiotics has declined markedly and drug-resistant bacteria are more common in hospitals and the community. In 2013, a report by the CDC reported that >2,000,000 people each year suffer from antibiotic resistant infections and >23,000 die as a result. Unfortunately, the number of new drugs to replace ineffective antibiotics is not adequate to meet current needs, and many major pharmaceutical companies have abandoned development of new antibiotics, focusing instead on new, long-term medications, such as statins and antihypertensives that produce greater profits. As an incentive for manufacturers to develop new antibiotics, in 2012, the Generating Antibiotic Incentives Now (GAIN) legislation was signed into US law as a part of the FDA Safety and Innovation Act. This legislation extends by 5 years the exclusivity period during which time those antibiotics that treat serious or life-threatening infections can be sold without generic competition. Drugs that fall under the GAIN provisions receive fast track and priority review status and undergo an expedited regulatory approval process with the FDA (137).

### Neonatal Gut Microbiome and Atopic Disease

New information from The Human Microbiome Project using cultivation-independent, molecular-based techniques has revolutionized our understanding of the vaginal microbiome in pregnancy and the non-pregnant state (38). The immune system is primed *in utero* and modified after birth. Accordingly, the use of antibiotics during pregnancy or the neonatal period may cause disruption of the developing neonatal gut microbiome, resulting in a failure of maturation of the immune response and the subsequent development of asthma, allergy, and atopic disease (138–141). This has led to new initiatives such as the Neomune Project publicly funded by the Danish Council for Strategic Research whose objective is to develop new diet and gut microflora treatments for new born infants.

## Evidence-Based Medicine

Guidelines issued by professional bodies, such as the Royal College of Obstetricians and Gynecologists, use a systematically developed standardized methodology (http://www.rcog.org.uk/guidelines) and a standardized grading scheme for the classification of evidence levels and grades of recommendations. Other organizations or governing bodies that produce guidelines use very similar methodology and schemes. The highest classification of evidence is 1++, which is defined as “high-quality meta-analyses, systematic reviews of randomized controlled trials or randomized controlled trials with a very low risk of bias.” The highest grade of recommendation is A which is defined as “At least one meta-analysis, systematic review or randomized controlled trial rated as 1++ and directly applicable to the target population; or a systematic review of randomized controlled trials or a body of evidence consisting principally of studies rated as 1+ directly applicable to the target population and demonstrating overall consistency of results.” Those who provide guidelines and recommendations base these on *some* SR&MAs of *some* RCTs as a result of which they may claim to be “evidence based.” SR&MAs are only as good as those studies included and if the questions asked about the populations, interventions, and outcomes are wrong or misdirected they should not be used to make recommendations or provide clinical guidelines (75). The Cochrane database is often the default of many clinicians looking for information, yet Cochrane is not without its faults, particularly in the area of PTB (142). Most SR&MAs of antibiotics for the prevention of PTB (Cochrane or otherwise) ask the question “In women at risk of PTB (population), do antibiotics (intervention) reduce the rate of PTB (outcome).” It is not surprising, therefore, that the updated Cochrane review of 2013 (74) contained data from 21 trials and required 57 different analyses because the included studies used different risk groups, diagnostic methods, degrees of abnormal microflora, antibiotic dose regimens and routes of administration, host susceptibilities, host response, gestational age at time of treatment, outcome parameters, and definitions of success (44–53, 55–58) resulting in different results. In contrast to other SR&MAs, in the AJOG SR&MA (43), the question was much more focused: “In pregnant women at risk of PTB of infectious etiology (population) does clindamycin administered before 22 weeks gestation (intervention) reduce the rate of PTB or late miscarriage (outcome).”

## Future Research

Even if the evidence based data is insufficient for some, they must at least accept that clinical equipoise exists (the ethical basis for medical research that involves assigning patients to different treatment arms of a clinical trial) and support a definitive randomized controlled trial. The choice of antibiotics (a) should be active against those organisms known to be associated with PTB, (b) should only be used in women with abnormal genital tract microflora, and (c) should be used early in pregnancy before infection and inflammation can cause irreversible tissue damage which will inevitably lead to SPTL and PTB. Such a study should contain genomic, transcriptomic, proteomic, and metabolomic studies to assess the vaginal microbiome, the vaginal milieu created by different microbiomic communities, and the host response of the individual to each sub-type of microbiome and milieu. PTB *per se* is only a surrogate for neonatal outcome. Detailed neonatal outcome data with appropriate long-term follow-up as well as the number of days gained from treatment to delivery should be the primary outcome parameters. Finally, different phenotypical outcomes of SPTL and PTB, such as late miscarriage, extreme PTB around the limits of viability, PPROM, late PTB, preterm stillbirth, and SPTL with intact membranes with or without vaginal bleeding should be considered. This is because the combination of different vaginal microbial communities, different vaginal milieu, and different host response may result in a range of phenotypic outcomes from normal term delivery to preterm stillbirth or severe morbidity associated with extremely premature birth.

## Conclusion

The earlier in pregnancy at which PTB occurs, the more likely this is to be due to infection (14). The earlier in pregnancy at which abnormal genital tract colonization is detected, the greater is the risk of an adverse outcome like late miscarriage or PTB (24). Abnormal vaginal microflora in early pregnancy, even if this resolves, is still associated with an adverse outcome (44) suggesting that whatever damage is caused by infection, this occurs early and persists. Accordingly, if antibiotics are to be used to prevent infection-related PTB these should be administered early. New evidence from molecular-based culture-independent studies of the vaginal microbiome (38) indicates that across the range of different microbial communities or sub-types of BV the bacteria detected are more likely to respond to clindamycin than metronidazole. Finally, treatment on the basis of the previous PTB should be predicated by some measure of infective etiology. Antibiotic treatment on the basis of previous PTB of unknown etiology or other risk factors for PTB unrelated to abnormal genital tract microflora should be discouraged. While individual studies have found benefit of antibiotic intervention for the prevention of PTB, in meta-analyses, these effects have been negated by large methodologically flawed studies with negative results. While (rightly) SR&MAs of efficacy focus on primary outcome parameters, the benefits associated with secondary outcomes are important and should not be ignored (*vide supra* Secondary outcomes) (43). At worst, equipoise exists with respect to the early use of clindamycin for the prevention of infection-related PTB. If a further, hopefully definitive trial is deemed necessary, this should be of a design and contains molecular omic data which will give a greater understanding of the underlying systems biology and mechanisms involved so that the same mistakes of previous flawed studies are not repeated. In the meantime, the use of antibiotics in pregnancy for the prevention of PTB should be restricted to those who are most likely to benefit.

## Conflict of Interest Statement

The author declares that the research was conducted in the absence of any commercial or financial relationships that could be construed as a potential conflict of interest.

## Abbreviations

AF, amniotic fluid; BMI, body mass index; BV, bacterial vaginosis; CDC, Centers for Disease Control and Prevention; CSAIDS, cytokine suppressive anti-inflammatory drugs; CVC, clindamycin vaginal cream; FDA, Food and drug Administration; GBS, group B streptococcus; HIV, human immunosuppressive virus; HPV, human papilloma virus; HSV, herpes simplex virus; IA, intra-amniotic; IL, interleukin; IV, intravenous; LPS, lipopolysaccharides; MMP, matrix metalloproteinases; PG, prostaglandin; PPROM, preterm prelabor rupture of the membranes; PTB, preterm birth; RCT, randomized controlled trial; SPTL, spontaneous preterm labor; SR&MA, systematic review and meta-analysis.
